# A national‐scale dataset for threats impacting Australia’s imperiled flora and fauna

**DOI:** 10.1002/ece3.7920

**Published:** 2021-08-04

**Authors:** Michelle Ward, Josie Carwardine, Chuan J. Yong, James E. M. Watson, Jennifer Silcock, Gary S. Taylor, Mark Lintermans, Graeme R. Gillespie, Stephen T. Garnett, John Woinarski, Reid Tingley, Rod J. Fensham, Conrad J. Hoskin, Harry B. Hines, J. Dale Roberts, Mark J. Kennard, Mark S. Harvey, David G. Chapple, April E. Reside

**Affiliations:** ^1^ Centre for Biodiversity and Conservation Science The University of Queensland St Lucia QLD Australia; ^2^ School of Earth and Environmental Sciences The University of Queensland Brisbane QLD Australia; ^3^ World Wide Fund for Nature‐Australia Brisbane QLD Australia; ^4^ CSIRO Land and Water Brisbane QLD Australia; ^5^ Department of Environment and Science Queensland Herbarium Brisbane QLD Australia; ^6^ School of Biological Sciences The University of Queensland Brisbane QLD Australia; ^7^ School of Biological Sciences Australian Centre for Evolutionary Biology and Biodiversity The University of Adelaide Adelaide SA Australia; ^8^ Centre for Applied Water Science University of Canberra Canberra ACT Australia; ^9^ Flora and Fauna Division Department of Environment, Parks and Water Security Northern Territory Palmerston SA Australia; ^10^ School of Biosciences University of Melbourne Melbourne VIC Australia; ^11^ Threatened Species Recovery Hub Research Institute for the Environment and Livelihoods Charles Darwin University Darwin NT Australia; ^12^ School of Biological Sciences Monash University Clayton VIC Australia; ^13^ College of Science & Engineering James Cook University Townsville QLD Australia; ^14^ Department of Environment and Science Queensland Parks and Wildlife Service and Partnerships Bellbowrie QLD Australia; ^15^ Biodiversity South Brisbane QLD Australia; ^16^ School of Biological Sciences The University of Western Australia Albany WA Australia; ^17^ Australian Rivers Institute Griffith University Nathan QLD Australia; ^18^ National Environmental Science Programme Northern Australia Environmental Resources Hub Darwin NT Australia; ^19^ Department of Terrestrial Zoology Western Australian Museum Weslshpool DC WA Australia

**Keywords:** Australian threatened species, EPBC Act, IUCN Threat Classification Scheme, IUCN Threat Impact Scoring System, Threat impacts, Threatened species

## Abstract

Australia is in the midst of an extinction crisis, having already lost 10% of terrestrial mammal fauna since European settlement and with hundreds of other species at high risk of extinction. The decline of the nation's biota is a result of an array of threatening processes; however, a comprehensive taxon‐specific understanding of threats and their relative impacts remains undocumented nationally. Using expert consultation, we compile the first complete, validated, and consistent taxon‐specific threat and impact dataset for all nationally listed threatened taxa in Australia. We confined our analysis to 1,795 terrestrial and aquatic taxa listed as threatened (Vulnerable, Endangered, or Critically Endangered) under Australian Commonwealth law. We engaged taxonomic experts to generate taxon‐specific threat and threat impact information to consistently apply the IUCN Threat Classification Scheme and Threat Impact Scoring System, as well as eight broad‐level threats and 51 subcategory threats, for all 1,795 threatened terrestrial and aquatic threatened taxa. This compilation produced 4,877 unique taxon–threat–impact combinations with the most frequently listed threats being *Habitat loss, fragmentation, and degradation* (*n* = 1,210 taxa), and *Invasive species and disease* (*n* = 966 taxa). Yet when only high‐impact threats or medium‐impact threats are considered, *Invasive species and disease* become the most prevalent threats. This dataset provides critical information for conservation action planning, national legislation and policy, and prioritizing investments in threatened species management and recovery.

## Graphical abstract

Australia is in the midst of an extinction crisis as a result of an array of threatening processes; however, a comprehensive taxon‐specific understanding of threats and their relative impacts remains undocumented nationally. Using expert consultation, we compile the first complete, validated, and consistent taxon‐specific threat and impact dataset for all 1,796 nationally listed threatened taxa in Australia. This compilation produced 4,877 unique taxon–threat combinations with the most frequently listed threats being Habitat loss, fragmentation, and degradation (*n* = 1,210 taxa), and Invasive species and disease (*n* = 966 taxa).

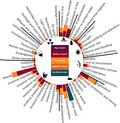

## 1 INTRODUCTION

The sixth mass extinction is arguably the worst environmental crisis humanity currently faces (Ceballos et al., 2020), with species becoming extinct 100–1,000 times faster than Earth's biota has experienced over the last ten million years (Barnosky et al., 2011; Ceballos et al., 2015; Pimm et al., 2014). Recent estimates show that one million species are now threatened with extinction (hereon “threatened”) globally and could go extinct in the next century (IPBES, 2018), with at least 515 terrestrial vertebrates likely to be lost within the next 20 years (Ceballos et al., 2020). In Australia, 25 taxa (ten birds, seven mammals, six reptiles, one butterfly, and twenty fish) are likely to become extinct within the next 20 years unless major conservation action is undertaken (“taxa” is used through the manuscript to collectively refer to species, subspecies, and important populations; Geyle, Braby, et al., 2021; Geyle, Tingley, et al., 2021; Geyle et al., 2018; Lintermans et al., 2020). This decline is driven by rapidly increasing direct and indirect pressures of human activities on species survival.

Australia is a large, sparsely populated continent that was geographically isolated until the late Miocene when biotic interchange with Asia commenced (Commonwealth of Australia, 2019; Woinarski et al., 2015). That isolation, coupled with harsh climates, rapid climate changes, and ca. 50,000 years of anthropogenically driven fire and hunting (Black et al., 2012; Crisp et al., 2011; Johnson, 2006; NSW Government, 2010; Wroe et al., 2013) has resulted in the unique evolution of biodiversity that is megadiverse and globally important (Black et al., 2012; Lindenmayer et al., 2010; Mittermeier & Mittermeier, 1997). Since 1788, European settlement has significantly changed the Australian environment by introducing novel species (e.g., woody and herbaceous weeds, cane toads, and cats; Lintermans et al., 2013; Woinarski et al., 2011), widespread clearing of native vegetation for intensive agriculture and urban development (Ward et al., 2019), ungulate grazing (e.g., sheep and cattle; Kuiper & Parker, 2013), spreading alien disease (e.g., *Phytophthora cinnamomi*, *Batrachochytrium dendrobatidis*; Skerratt et al., 2007), and altering fire regimes (Woinarski et al., 2015). These changes have resulted in threatening processes that have an especially profound impact on native species. However, the state of knowledge of the most important threats and threat impacts responsible for the declines and extinctions is fundamentally lacking.

Previous efforts to assess threats to Australia's Environment Protection and Biodiversity Conservation (EPBC) Act (1999) listed threatened species include the Australian Government's Species Profiles and Threats Database (hereafter “SPRAT”; Allek et al., 2018; Commonwealth of Australia, 2021a; Kearney et al., 2019), where “invasive species and disease” is listed as the most prevalent of a set of key threats impacting on nationally threatened Australian fauna and flora (Allek et al., 2018; Kearney et al., 2019, 2020). However, the SPRAT dataset does not address habitat loss, fragmentation, and degradation as a threat, nor include the most up‐to‐date knowledge on the level of impact each threat has on each taxon. This more detailed knowledge held by relevant experts has, until now, been uncollated or undocumented at a national‐scale. Consequently, policy‐makers, decision‐makers, and practitioners are unable to access a comprehensive dataset of taxon‐specific threats, including information that systematically differentiates between negligible threats from those that cause significant, catastrophic declines over contemporary time periods (Cross et al., 2019).

Australia requires an improved dataset that identifies the importance of different threats at the taxonomic level at which the entity is listed as threatened. The IUCN’s Threats Classification Scheme (IUCN, 2015; Salafsky, 2008) and Threat Impact Scoring System (IUCN, 2012a) are globally recognized approaches for classifying threats and ranking the level of impact each threat has on specific species (IUCN, 2012a). The IUCN Threat Impact Scoring System includes information on the timing of the threat, the proportion of the total population affected, and the overall declines caused by the threat. This method has been applied to IUCN Red List assessments of some species globally, including Australian species such as koala (*Phascolarctos cinereus*), quokka (*Setonix brachyurus*), freshwater fishes, and all Australian birds (Birdlife International, 2018; Brooks et al., 2019; Burbidge & Woinarski, 2020; Garnett et al., 2019; Lintermans & Allan, 2019; Woinarski & Burbidge, 2020), but not yet comprehensively for all threatened taxa.

Here, we engaged taxonomic experts in generating taxon‐specific threat and threat impact information to consistently apply the IUCN Threat Classification Scheme and Threat Impact Scoring System to produce the most up‐to‐date data on currently recognized threatening processes affecting all nationally listed threatened taxa in Australia. We produced a comprehensive taxon–threat–impact dataset that identifies all IUCN threat types and detailed threat notes, in addition to eight new broad‐level threats and 51 subcategory threats, for all 1,795 threatened terrestrial and aquatic threatened taxa. We created this novel categorization based on extensive discussion with experts and managers, which draws heavily upon existing categories but is modified in order to have a classification that was fit to the Australian context of threats, governance of threatened species recovery, and threat abatement planning. The categories can also be used for communicating the major causes of threatened species decline to a range of audiences. In total, our dataset contains 4,877 taxon–threat–impact combinations, which includes timing, scope, and severity for all combinations, where available. This information will allow for comprehensive, consistent, national‐scale assessment of taxon‐specific threatening processes and their degree of impact, to guide appropriate conservation actions that will facilitate taxa to persist and recover in the future.

## 2 MATERIALS AND METHODS

### 2.1 Threatened taxa in Australia

Under Australia's *EPBC Act* 1999, there are six categories of threat status: Extinct, Extinct in the Wild, Critically Endangered, Endangered, Vulnerable, and Conservation Dependent. We confined our analysis to 1,795 terrestrial and aquatic taxa listed as threatened (Vulnerable, Endangered, Critically Endangered, or Extinct in the Wild) under Australia's EPBC Act as of July 2018. We excluded taxa that were listed as Extinct or Conservation Dependent (the latter pertaining only to commercially harvested fish taxa that have a specific conservation program; however, the cessation of which would result in the species becoming Vulnerable, Endangered, or Critically Endangered). For taxa that are not endemic to Australia, information was compiled on all threatening processes.

### 2.2 Knowledge synthesis process

To synthesize knowledge and collate the taxon–threat–impact dataset, we followed five key steps: (i) identifying key data needs; (ii) designing and preparing the expert assessment; (iii) implementing the expert consultation (Hadwen et al., 2011; Pullin et al., 2016); (iv) encoding the expert responses; and (v) completing a technical validation. The expert consultation process was carried out from December 2019 to September 2020. As facilitators of the assessment process, we emailed fourteen experts to first describe the data required (i.e., threats and threat impact scores per taxon), provide instructions for the assessment, and distribute datasheets required for the assessment. Experts were chosen based on their extensive expertise in taxon groups, of which many had already begun the process of consolidating information on threats for their respective taxa of interest. The experts then consulted with relevant colleagues and searched existing literature to identify and complete the dataset (see Appendix S1) for taxon‐specific threats and the components of each threat needed to estimate its likely impact using timing, scope, and the overall severity of the threat. In some cases, full systematic Conservation Action Planning workshops were completed for individual taxon to detail their threats and the likely impact of each (Black‐throated Finch Recovery Team, 2020). The overall threat impact is then classified as high, medium, low, negligible, or insufficient data (i.e., missing values from timing, scope, and severity) using the IUCN Threat Impact Scoring System (Garnett et al., 2019; IUCN, 2012a). Once the information was received and reviewed, follow‐up consultations were conducted with the lead experts to resolve any uncertainty and seek additional clarification regarding specific threats. Facilitators then encoded the expert's responses resulting in a consistent, comprehensive list of all threats and the impact of each threat to every taxon, where knowledge was available. The dataset was encoded to include the IUCN threat categories (variable name: *IUCN threat level 1*, *IUCN threat level 1 description*, *IUCN threat level 2*, *IUCN threat level 2 description*, *IUCN threat level 3*, and *IUCN threat level 3 description*), eight broad‐level threat categories, and 51 subcategory threats (variable name: *Broad‐level threats, Subcategory threats*; Table 1). The additional broad‐level threats and subcategory threats were necessary as the IUCN threat categories failed to capture some threats that Australian taxa are exposed to, including *Habitat loss, fragmentation, and degradation,* and *Disrupted ecosystem and population processes*. The threat categories developed here deviate from the IUCN approach in an effort to identify what threats taxa experience (e.g., habitat loss, degradation, and fragmentation) as well as the ultimate cause of those threats (e.g., housing development). These categories also allow a threatened species manager to understand the direct threat to the species and hopefully have more information on actions. For example, a biodiversity officer in a state government likely cannot do much about a climate change resulting in habitat alteration, but might be more equipped to address habitat loss, degradation, and fragmentation. While the IUCN does provide a Stresses Classification Scheme (IUCN, 2012b), we found that these categories were not fit for our purpose. For example, ecosystem conversion and ecosystem degradation are usually inextricably linked; and in many cases, species are impacted by both. In addition, we required a classification which linked the threat to an action. If the same threat stresses two species differently, the threat abatement at a high level would remain the same. Therefore, for this research, it was better to focus on using threats that could be more easily linked to threat abatement actions. These categories were discussed and decided upon during three workshops held from July to August 2020 with independent experts from the Australian Threatened Species Scientific Committee (TSSC) (Commonwealth of Australia, 2020) and close collaborators of the TSSC. During these workshops, participants used relevant literature (Cattarino et al., 2018; Kearney et al., 2020) to help guide discussion and decide upon Australian‐specific broad‐level and subcategory threats.

**TABLE 1:** The eight broad‐level threat categories and 51 subcategory threats used in the Australia‐wide analysis on what threatening processes impact threatened taxa. The symbols are used in Figure 2

Broad‐level threats	Symbol	Subcategory threats
Adverse fire regimes	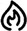	Increase in fire frequency/intensity Suppression in fire frequency/intensity Other change in fire regime/trend unspecified
Changed surface and groundwater regimes	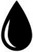	Alteration to groundwater levels Alteration to surface water flows and infiltration Dams and altered flow regimes
Climate change and severe weather	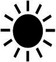	Climate change and severe weather‐unspecified Habitat shifting and alteration Increased frequency/severity of droughts Sea‐level rise Storms and flooding Temperature extremes
Disrupted ecosystem and population processes	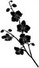	Genetic introgression/hybridization Lack of recruitment Problematic native species Small, restricted, and reduced population
Habitat loss, fragmentation, and degradation	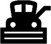	Agriculture and aquaculture Energy production and mining Fisheries Forestry Geological events Military development Transportation and service corridors Urban and commercial development and maintenance Other natural system modifications
Invasive species and diseases	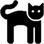	Disease Invasive amphibian Invasive bird Invasive fish Invasive invertebrate Invasive predator Invasive rabbit Invasive reptile Invasive rodent Invasive ungulate Invasive weed
Overexploitation and other direct harm from human activities	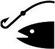	Collision Direct harvest Human intrusion Persecution Unintentional poisoning Unintentional hunting Entanglement Bycatch
Pollution	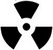	Effluent and wastewater Garbage and solid waste Herbicides and pesticides Light pollution Nutrient loads Oil spills Seepage from mining

### 2.3 IUCN Threat Impact Scoring System

The IUCN Threat Impact Scoring System (Table 2) scores threats to a taxon based on the timing of the threat (i.e., past, ongoing, future), the scope of the threat (defined as the proportion of the whole population affected), and severity of the threat on the taxon (i.e., the overall declines caused by the threat; Garnett et al., 2019; IUCN, 2012a). The IUCN threat impact scores are summed to provide the overall threat impact (based on IUCN 2012; IUCN, 2012a; Table 3). For example, Mary River Cod (*Maccullochella mariensis)* is threatened by fishing and harvesting, which is an ongoing (timing = 3) threat, affecting the whole population (scope = 3), and causes slow, but significant declines (severity = 1). The overall impact is 7, resulting in an overall impact score of “medium.”

**TABLE 2:** IUCN Threat Impact Scoring System (based on IUCN, 2012a) applied in the Australia‐wide analysis on threatening processes impacting threatened taxa

Criteria	Categories and scores
**Timing**	
Only in the past and unlikely to return	0
In the past but now suspended and likely to return	0
Ongoing	3
Only in the future	1
Unknown	0
**Scope**	
Affects the whole population (>90%)	3
Affects the majority of the population (50%–90%)	2
Affects the minority of the population (<50%)	1
Unknown	0
**Severity**	
Causing or likely to cause very rapid declines (>30% over 10 years or three generations, whichever is longer)	3
Causing or likely to cause rapid declines (20%–30% over 10 years or three generations, whichever is longer)	2
Causing or likely to cause relatively slow but significant declines (<20% over 10 years or three generations, whichever is longer)	1
Causing or likely to cause fluctuations	1
Causing or likely to cause negligible declines	0
No declines	0
Unknown	0

**TABLE 3:**
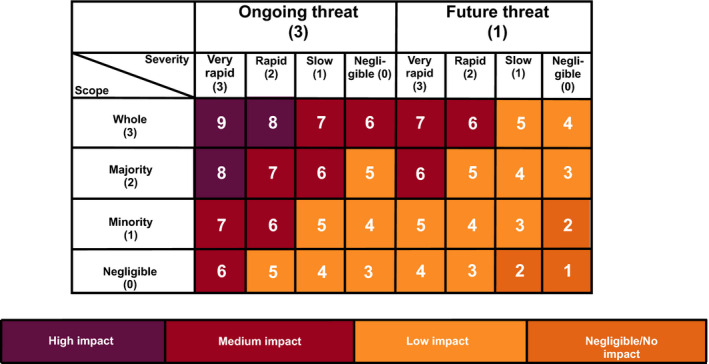
IUCN threat impact scores where timing, scope, and severity are summed (based on IUCN, 2012a). Relative levels of impact are color‐coded as dark purple (high impact), maroon (medium impact), tangarine (low impact), and bronze (negligible impact)

Experts were provided with datasheets that elicited their estimates of scope, severity, and timing. The overall threat impact scores were automatically calculated in the datasheet based on predefined IUCN thresholds driven by the summed value of the timing, scope, and severity scores (>7 = high impact, >5 = medium impact, >2 = low impact, and >0 = negligible impact). Some taxonomic groups had existing information that was included in the datasheets before they were sent to experts (Table 4).

**TABLE 4:** Existing threat data used in the data collation process to assist in synthesizing and formulating the taxa–threat–impact dataset

Taxonomic group	Experts	Data incorporated
Mammals	John Woinarski, Andrew Burbidge	Woinarski et al. (2014) comprehensively reviewed the conservation status of all Australian mammals. We used this dataset to initially describe the threats and scores based on their scoring method. These threats and impact scores were then verified by experts during the elicitation process (Lumsden & Jemison, 2015).
Birds	Stephen Garnett	Garnett et al. (2019) and Garnett and Baker (2021) provided data for each threatened Australian bird taxon, threats, and threat scores which were directly embedded within this dataset. The original Garnett et al. (2019) bird datasets contain 244 taxa (118 from the 2020 dataset and 126 from the 2019 dataset). Of the 135 nonextinct EPBC‐listed bird taxa, 57 had updated data from the 2020 assessment (Garnett & Baker, 2021); and data for the remaining 78 bird taxa came from Garnett et al. (2019). These threats and impact scores were verified by experts during the expert consultation process (Department of the Environment, 2013).
Reptiles	Reid Tingley, David Chapple	We incorporated all data from Tingley et al. (2019) and Chapple et al. (2017), who identified all threatening processes impacting Australian squamates. These threats and impact scores were directly embedded within this dataset and then verified by experts during the expert consultation process. Data for all other reptile taxa were gathered during the expert consultation (Legge et al., 2019; Woinarski et al., 2014).
Frogs	Graeme Gillespie, David Hunter, Conrad Hoskin, Harry Hines, Dale Roberts	Existing data for Australian frogs (Gillespie et al., 2020; Heatwole & Rowley, 2018) were incorporated and additional threat impact information was elicited from relevant experts (Garnett & Baker, 2021; Tingley et al., 2019).
Fish	Mark Lintermans, Mark Kennard, Helene Marsh, Colin Simpfendorfer, and Lesley Gidding‐Reeve	Data for Australian threatened freshwater taxa from existing threat assessments was incorporated (e.g., Lintermans, 2013 and Lintermans et al., 2013). Additional threat impact information was sourced from the 2019 freshwater and marine Red List assessment and elicited from relevant experts (Chapple et al., 2017).
Invertebrates	Gary Taylor	While there are existing data (Taylor et al., 2018) for Australian threatened invertebrates, additional threat impact information was required for data consistency. Therefore, the expert elicitation process outlined above was undertaken. Existing data for threats to EPBC‐listed invertebrates (Heatwole & Rowley, 2018) were guided by threat impacts identified in their EPBC listing and IUCN red list (Lintermans, 2013) (not exhaustive, restricted to the perceived main threats), and supplemented with data from expert consultation process (Gillespie et al., 2020).
Plants	Jennifer Silcock, Rod Fensham	Existing data for threats to EPBC‐listed plants (Silcock & Fensham (2018) and Silcock et al., 2020) were supplemented with data from expert elicitation (Commonwealth of Australia, 2021b; Taylor et al., 2018).

### 2.4 Technical validation

We developed the final dataset in R (version 1.2.5033), which encompassed a validation process. This validation process was undertaken by each of the expert teams by cross‐checking threat categories (IUCN, broad‐level, and subcategories), threat codes, and threat impact scores, taxonomy, and standardizing taxon names and threat statuses.

## 3 RESULTS

### 3.1 Australia's threatened taxa

Of all the EPBC Act listed threatened taxa in Australia, plants are the numerically dominant threatened group (74.6%), yet only 7.2% of 18,706 accepted/described plants in Australia are threatened. Mammals represent only 6% of all listed threatened taxa, yet c.28% of all Australian mammals are listed as threatened (Table 5). On average, each taxon was threatened by three subcategory threats (median = 2; range = 1–15).

**TABLE 5:** Overview of the number of threatened taxa per group within Australia, proportion of threatened taxa within each group out of the total number of threatened taxa in Australia, and proportion of threatened taxa within each group out of the total taxa in each group within Australia (Chapman, 2009; Commonwealth of Australia, 2021a)

Group	No. of threatened taxa	% of total threatened taxa	No. of taxa in Australia	% of group listed as threatened
Plants	1,339	74.6%	18,706	7.2%
Birds	135	7.5%	828	16.3%
Mammals	107	6.0%	386	27.7%
Invertebrates	65	3.6%	320,000	0.02%
Reptiles	61	3.4%	917	6.6%
Fish	51	2.8%	5,000 (or 315 freshwater fish)	1.0% (or 12% of freshwater fish)
Frogs	37	2.1%	227	16.3%
**Total**	**1,795**			

### 3.2 Broad‐level and subcategory threatening processes

**FIGURE 1:**
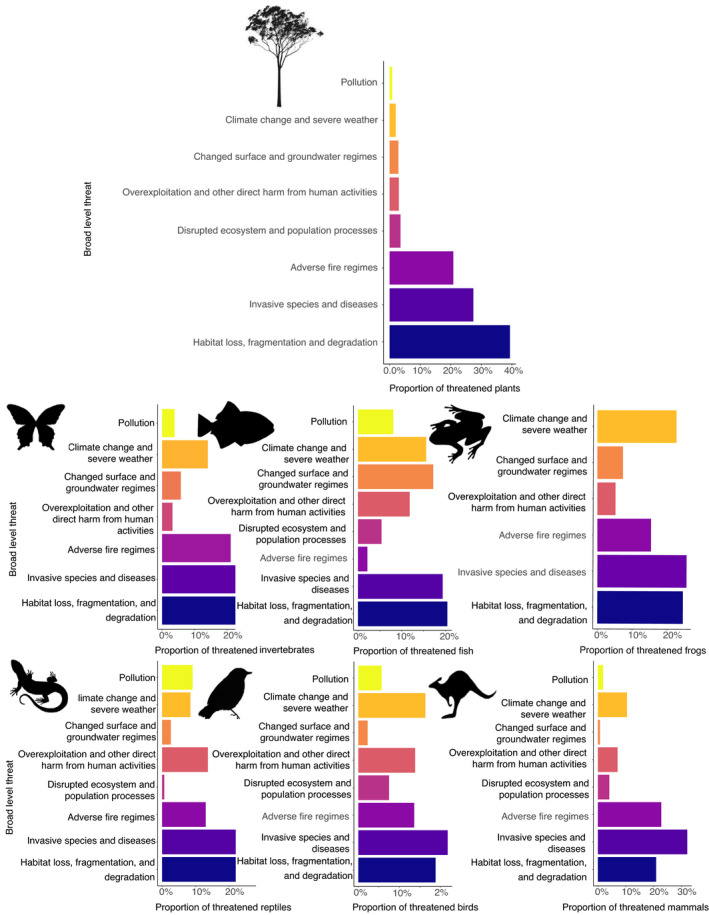
Proportion of Australian threatened taxa impacted by broad‐level threats. Each bar chart represents a different group, including plants, invertebrates, fish, frogs, reptiles, birds, and mammals. Threats including *Habitat loss, fragmentation, and degradation* (dark blue), *Invasive species and disease* (indigo), *Adverse fire regimes* (purple), *Disrupted ecosystem and population processes* (magenta), *Overexploitation and other direct harm from human activities* (coral), *Changed surface and groundwater regimes* (orange), *Climate change and severe weather* (gold), and *Pollution* (yellow)

Our investigation summarizes threats using eight broad‐level threats and 51 sub‐category threats that together impact upon 1,795 terrestrial and aquatic taxa, totaling 4,877 unique taxon–threat combinations. The most frequently listed broad‐level threats were *Habitat loss, fragmentation, and degradation* (*n* = 1,210 taxa), *Invasive species and diseases* (*n* = 966 taxa), and *Adverse fire regimes* (*n* = 683 taxa). However, different taxonomic groups are threatened by different pressures (Figure 1). For example, while *Habitat loss, fragmentation, and degradation* is the key threatening process for invertebrates, fish, reptiles, and plants, *Invasive species and diseases* threaten the most birds, frogs, and mammals.

**FIGURE 2:**
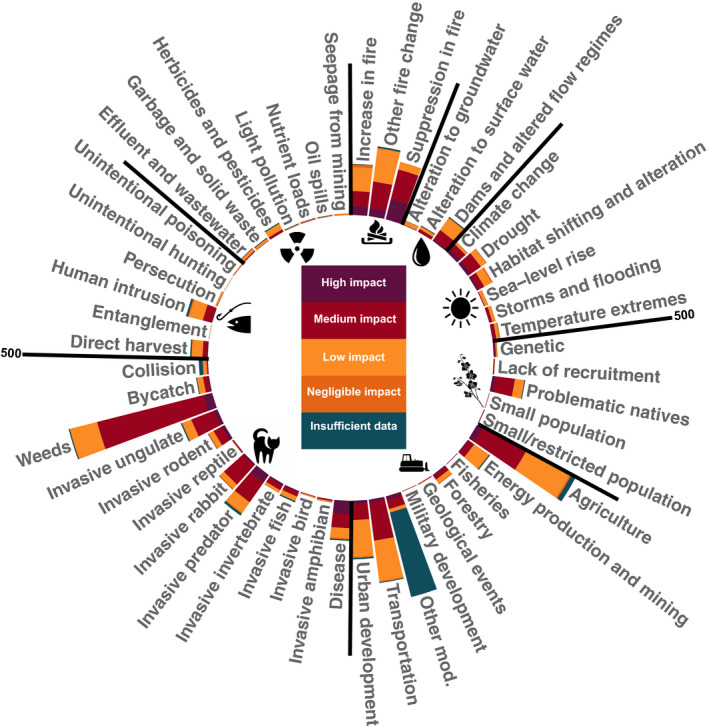
Number of threatened Australian taxa and relative level of impact for each subcategory threat, nested within the corresponding broad‐level threat class. See Table 2 for symbols representing each broad‐level threat. Relative levels of impact are color‐coded as dark purple (high impact), maroon (medium impact), tangarine (low impact), bronze (negligible impact), and teal (insufficient data). The scale bar indicates the cumulative number of taxa impacted per threat

Examination of the subcategory threats can aid understanding of the main causes of each broad‐level threat within which it is nested (Figure 2). The most frequently listed subcategory‐level threats were *Invasive weeds* (nested within *Invasive species and disease* with *n* = 565 taxa), *Agriculture and aquaculture* (nested within *Habitat loss, fragmentation, and degradation* with *n* = 411 taxa), and *Other natural system modifications* (also nested within *Habitat loss, fragmentation, and degradation n* = 398 taxa).

### 3.3 Impact of threats across taxa

**FIGURE 3:**
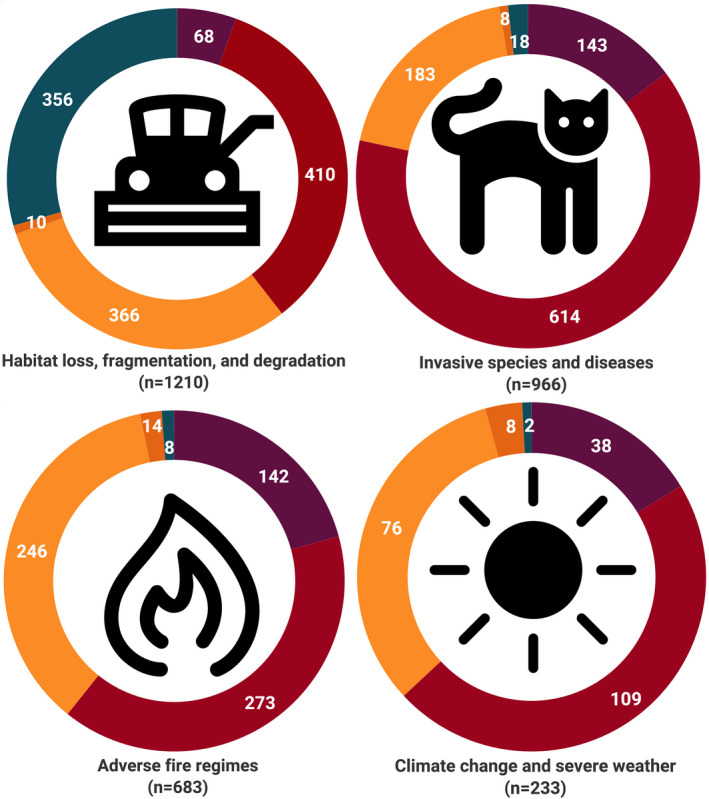
The most important threats to threatened Australian taxa change when impact is considered. The diagrams show the number of taxa per impact score within the broad‐level threat (a) *Habitat loss, fragmentation, and degradation*, (b) *Invasive species and diseases*, (c) *Adverse fire regimes*, and (d) *Climate change and severe weather*. Impact was determined through the evaluation of timing, severity, and scope for each threat per taxon. Where a taxon was threatened by multiple subcategories within a broad threat, we used the maximum impacting level in this analysis. For example, if a taxon was assessed as being threatened by *Residential and commercial development* at a low impact and *Agriculture and aquaculture* at a high impact under the IUCN classification scheme, which both fall under the broad‐level threat of *Habitat loss, fragmentation, and degradation*, the broad‐level threat was considered high impact for that taxa

The ranking of threats changes when the impact of the broad‐level threat is considered (Figure 3). When only high‐impact or medium‐impact threats are considered, *Invasive species and diseases* (*n* = 143 taxa and *n* = 614 taxa, respectively) become the key threats to taxa compared to *Habitat loss, fragmentation, and degradation* (*n* = 68 taxa and *n* = 410 taxa, respectively). For 9.6% (*n* = 464) of taxon–threat combinations, impact scores were unattainable due to insufficient data, which appear to be associated with a lack of understanding of the level of impact that habitat modifications have on threatened species. This outcome reflects the reality of complex threatening processes and critical knowledge gaps concerning threats to Australia's threatened biodiversity, where experts are able to identify a possible threat but are not able to confidently evaluate the degree of impact it has on a particular taxon.

## 4 DISCUSSION

Our results build on other global and continental analyses that have explored which threatening processes affect most taxa. Global analyses have revealed overexploitation as the prevalent threatening process (Maxwell et al., 2016; Yiming & Wilcove, 2005), but across Australia, we show that mitigating the impacts of habitat loss, fragmentation, and degradation will benefit the greatest number of taxa overall. Since 2000, 85% of Australia's threatened species lost habitat, equating to 7.7 million hectares, and efforts to ameliorate this ongoing loss have had little effect (Ward et al., 2019). As habitat loss is primarily driven by agriculture and urban development (Evans, 2016), it is a politically polarizing issue (Lindenmayer et al., 2014). However, habitat is the most fundamental need of species, and its continued loss will result in ongoing declines regardless of how well other threats are managed. Threats such as invasive species are also severely affecting Australian threatened taxa, despite many initiatives aimed at reducing their impacts; for example, Non‐Governmental Organisations and Commonwealth and state governments have invested heavily in the creation of predator‐proof refuges and managing feral cats at various geographical scales via massive baiting efforts (Commonwealth of Australia, 2014; Department of the Environment, 2015). Our dataset shows that mitigating habitat loss, invasive species, and disease, along with improving fire regimes, and where possible, adaptation to climate change, is crucial for curbing species declines.

We anticipate this dataset will provide critical information to help inform conservation and management strategies for Australia's threatened species and threatening processes at local, regional, and national scales. For example, when used in combination with other key climate information, this dataset could assist in guiding action to build species resilience in the face of climate change and other related catastrophic events, such as the 2019–2020 megafires (Legge et al., 2020; Ward et al., 2020). Our dataset can help guide actions for abating existing threats to bushfire‐impacted species to help aid recovery and avoid further declines. This taxon–threat–impact dataset can also be used to infer the benefit of managing a particular threat and aid in recovery planning (Cattarino et al., 2015, 2018). For example, the Endangered south‐eastern subspecies of the Spot‐tailed Quoll (*Dasyurus maculatus maculatus*) has 12 recorded threats, one of which is considered to be of high impact, two are of medium impact, and nine are of low impact. This indicates that while the one high‐impacting threat, invasive foxes, is a high priority for mitigation, lower impacting threats such as cane toads and mortality associated with road traffic are likely to be lower priorities for mitigation. The dataset may be used at the local scale, where decision‐makers can use the severity score to decide which of the threats present in their jurisdiction are the most important and feasible to address. Another example might be Southern Bent‐wing Bat (*Miniopterus orianae bassanii*), which is threatened by human intrusion. This threat is continuing (timing = 3), primarily problematic in maternity caves (scope = 1), and can cause very rapid declines (severity = 3). Therefore, while the scope is low, the overall impact of human intrusion is medium, and managers of these important roosts (e.g., Warrnambool City Council and Naracoorte Lucindale Council) may decide to prioritize protecting these roosts from human disturbance (Lumsden & Jemison, 2015). This dataset can also be used to refine regulatory processes given the level of impact to particular taxa. For example, under the EPBC Act, actions associated with a particular development proposal or other activities that are likely to cause “significant impact” to a threatened taxon require special consideration (Department of the Environment, 2013). This dataset may aid decision‐makers in determining “significant impact” of potential activities for each of Australia's nationally listed threatened taxa. Our results highlight the urgent need to address the many high‐ and medium‐impact threats, the majority of which consisted of invasive species and diseases and habitat loss, fragmentation, and degradation. This newly collated, consistent, national‐scale information contributes to taxon‐specific or threat‐specific assessment to guide appropriate conservation actions that will facilitate taxa to persist and recover in the future.

A limitation of this taxon–threat–impact dataset is that it only integrates historic and recent information up to present day. This dataset therefore cannot be used to assess the impacts of changes in threat exposure and intensity over time, but we hope future revisions of the dataset will enable this. Being national in scale means that spatially variable differences or threats from other countries have also not been considered. Interactions among threats are not specifically considered, but there is increasing evidence of cumulative and synergistic impacts of co‐occurring and interacting threats (Legge et al., 2019). A further limitation is that the dataset focuses on nationally listed taxa as of 2018 and many taxa potentially eligible for listing are currently unlisted (e.g., Lintermans et al., 2020), and this number is likely to increase as Australia’ biota experiences broad‐scale catastrophic events such as the 2019–2020 bushfires (Evans, 2016). Therefore, there are likely to be many taxa threatened with extinction for which management efforts, such as legislative instruments, to mitigate threats are currently nonexistent. While this is the most up‐to‐date data available, there are several threats such as anthropogenic‐driven climate change resulting in adverse fire regimes, increased droughts, spreading invasive species, and range shifts that are expected to worsen in impact and threaten more species than are currently listed. Such emerging threats must be incorporated in future iterations of this threat analysis. It is our vision that this dataset will periodically be updated and improved. We recommend that the most reliable way for this dataset to be maintained and sustained is to tie it to the formal EPBC Act assessment process.

## CONFLICT OF INTEREST

The authors declare no competing interests.

## AUTHOR CONTRIBUTION

**Michelle Ward:** Conceptualization (equal); Formal analysis (equal); Investigation (equal); Methodology (equal); Project administration (equal); Validation (equal); Visualization (equal); Writing‐original draft (equal); Writing‐review & editing (equal). **Josie Carwardine:** Formal analysis (equal); Writing‐review & editing (equal). **Chuan J. Yong:** Validation (equal); Writing‐review & editing (equal). **James E. M. Watson:** Conceptualization (equal); Supervision (equal); Visualization (equal); Writing‐review & editing (equal). **Jennifer Silcock:** Data curation (equal); Validation (equal); Writing‐review & editing (equal). **Gary S. Taylor:** Data curation (equal); Writing‐review & editing (equal). **Mark Lintermans:** Data curation (equal); Writing‐review & editing (equal). **Graeme R. Gillespie:** Data curation (equal); Writing‐review & editing (equal). **Stephen T. Garnett:** Data curation (equal); Methodology (equal); Writing‐review & editing (equal). **John Woinarski:** Data curation (equal); Writing‐review & editing (equal). **Reid Tingley:** Data curation (equal); Writing‐review & editing (equal). **Rod J. Fensham:** Data curation (equal). **Conrad J. Hoskin:** Data curation (equal); Writing‐review & editing (equal). **Harry B. Hines:** Validation (equal); Writing‐review & editing (equal). **J. Dale Roberts:** Data curation (equal); Writing‐review & editing (equal). **Mark J. Kennard:** Validation (equal); Writing‐review & editing (equal). **Mark S. Harvey:** Data curation (equal); Writing‐review & editing (equal). **David G. Chapple:** Data curation (equal); Writing‐review & editing (equal). **April E. Reside:** Formal analysis (equal); Supervision (equal); Writing‐review & editing (equal).

### OPEN RESEARCH BADGES

This article has earned an Open Data Badge for making publicly available the digitally‐shareable data necessary to reproduce the reported results. The data is available at https://doi.org/10.6084/m9.figshare.13150943.v1

## Supporting information

Appendix S1

## Data Availability

The taxon–threat–impact dataset is available in the Appendix S1 or via Figshare (https://doi.org/10.6084/m9.figshare.13150943.v1). Data for each group of taxa (i.e., mammals, birds, reptiles, frogs, invertebrates, plants, and fish) are also provided in the data record to enable group‐specific interrogation of information.
